# The Prevalence of Severe Visual Impairment and Blindness Among Primary School Children in Arar, Saudi Arabia

**DOI:** 10.7759/cureus.47833

**Published:** 2023-10-27

**Authors:** Basem Salama, Mujeeb Ur Rehman Parrey, Maha M Abdul-Latif, Hussam M Alanazi, Saja R Alanazi

**Affiliations:** 1 Community Medicine, Northern Border University, Arar, SAU; 2 Surgery, Northern Border University, Arar, SAU; 3 College of Medicine, Northern Border University, Arar, SAU

**Keywords:** visual impairment, saudi arabia, prevalence, northern border university, blindness

## Abstract

Background

Childhood visual impairment has a major impact on the growth and development of children at all levels. The affected children are likely to experience psychological issues and less successful social integration. Besides healthcare providers, researchers, teachers, and parents can play a vital role in reducing the burden of this global health issue.

Methodology

This cross-sectional study was conducted among children attending all primary schools in Arar to measure the prevalence of severe visual impairment and blindness. The uncorrected and best-corrected visual acuity was assessed and the prevalence of visual impairment was determined in line with the WHO guidelines.

Results

A total of 781 Saudi children aged between six and 12 years participated in this study. These included 403 (51.6%) females and 378 (48.4%) males. The prevalence of severe visual impairment and blindness according to WHO classification was found in 14 and seven children, respectively.

Conclusions

The prevalence of severe visual impairment and blindness in primary school children in Arar is high. The burden of visual impairment can be reduced by improving the approach toward early screenings and timely addressing the factors associated with such visual impairment in children.

## Introduction

When the visual pathway’s function is changed, vision is said to be impaired. Visual impairment (VI) is assessed by visual acuity (VA) testing and the VA achieved after correction. Based on the WHO classification, VA equal to or more than 6/18 is considered as normal vision, less than 6/18 but more than 6/60 as VI, and less than 6/60 but more than 3/60 as severe VI [1]. Blindness is defined as best-corrected VA in the better eye being less than 3/60 [2].

An estimated 36 million people worldwide are blind [3]. According to the prevalence of blindness by age distribution, there are currently 17.5 million people who are at risk of having low vision compared to 1.4 million children aged below 14 years who are blind [4]. The burden of childhood blindness is thought to be 70 million blind person-years [5]. In developing countries, childhood blindness of up to 31% can be prevented, up to 58% can be treated, and up to 28% can be avoided [6].

While in developed countries unavoidable congenital causes are more commonly responsible for VI, in less developed nations, VI has more preventable causes [7].

The journey of learning starts in early childhood and a child’s ability to learn is significantly influenced by the level of their VA. When a child suffers from VI, their social interaction, growth, and academic performance can be remarkably affected [8,9]. Besides, children who are visually handicapped or suffer from blindness contribute to a higher economic burden on the family and society [10].

All children should get inclusive, equitable, and high-quality education, according to Sustainable Development Goal 4. However, because vision is important for a child’s learning and development, uncorrected refractive errors have been a significant barrier to providing them with a proper education [11].

To promote students’ health, the WHO started the Global School Health Initiative in 1995 and advised screening for visual difficulties in children and offering them refractive therapies in schools [12].

Active screening offers data on the prevalence, distribution, and causes of refractive error, which is required to plan and deliver eye care services properly. Well-planned earlier intervention helps in vision restoration with a positive impact on a child’s growth and development. Therefore, this study aimed to measure the prevalence of severe VI and blindness among primary school children in Arar.

## Materials and methods

Study design and location

This cross-sectional study was conducted from August 2022 to May 2023 among 782 primary school children. The study was conducted in Arar, the capital city of the Northern Border Region of Saudi Arabia.

Inclusion and exclusion criteria

All primary school children aged between six and 12 years who agreed to participate were included and those who refused to participate were excluded from this study.

Ethical approval

Ethical approval was obtained from the Scientific Research Committee of Northern Border University (approval number: HAP-09-A-043). Informed consent was obtained from the parents/guardians of each participant.

Methodology

Distant VA, in each eye separately, was tested on Snellen’s VA chart placed at 6 m in a well-illuminated area. The students were initially screened at the schools and those found to have reduced VA were further evaluated at the University Health Centre. One ophthalmologist and one optometrist conducted a complete eye examination of these children. We determined severe visual impairment (SVI) as VA less than 6/60 but more than 3/60 and blindness as best-corrected VA in the better eye being less than 3/60 as per the WHO criteria. [1] The minimum sample size of 384 children was calculated using Epi Info (version 7.1.5, 2015) based on the assumption that the VI was 50%, the precision was 5%, and 95% confidence level.

Statistical analysis

Depending on the type of each item, the data are reported as frequencies, proportions, or mean and standard deviations and were analyzed using SPSS (IBM Corp., Armonk, NY, USA). The association between two categorical variables was assessed using the chi-square test. Logistic regression analysis was applied to determine the independent risk factors. At 95% confidence intervals, statistically significant differences were defined as p-values of 0.05.

## Results

A total of 781 Saudi children participated in this study. These included 403 (51.6%) females and 378 (48.4%) males. The ages of the children ranged from six to 12 years with a mean of 10 ± 2.9 years. The demographic details of all participants are shown in Table 1.

**Table 1:** Demographic data of the participants (total participants = 781).

Parameter		Number	%
Sex	Female	403	51.6
	Male	378	48.4
Age (years)	6–9	252	32.27
	10–12	529	67.73
Father’s education	Below higher education	310	39.69
	Higher education	471	60.31
Mother’s education	Illiterate	44	5.63
	Below higher education	445	56.98
	Higher education	292	37.39
Family history of eye diseases	Neither	546	69.91
	Father	154	19.72
	Mother	73	9.35
	Both	8	1.02
Frequent eye inflammation	Yes	283	36.24
	No	498	63.76
Socioeconomic status	Sufficient	774	99.10
	Insufficient	7	0.90
Physical activity	<2 hours/day	606	77.59
	>2 hours/day	176	22.54
School type	Private	52	6.66
	Governmental	729	93.34
Smartphone use	<2 hours/day	61	7.81
	2–4 hours/day	334	42.77
	>4 hours/day	386	49.42

This study revealed that according to the WHO classification, 760 (97.2%) had no SVI or blindness. Severe VI was found in 14 (1.8%) children while blindness was seen in seven (0.9%) children. The status of VI among the studied participants is described in Table 2.

**Table 2:** Status of VI in the studied participants. BL = blindness; VI = visual impairment; SVI = severe visual impairment

Total participants = 781		No SVI/BL		SVI		BL	
		Number	%	Number	%	Number	%
		760	97.2	14	1.8	7	0.9
Sex	Female	374	49.2	11	78.6	4	57.1
	Male	386	50.8	4	28.6	3	42.9
Age (years)	6–9	247	32.5	6	42.9	2	28.6
	10–12	520	67.5	8	57.1	5	71.4
Father’s education	Below higher education	283	37.2	11	78.6	2	28.6
	Higher education	477	62.8	3	21.4	5	71.4
Mother’s education	Illiterate	36	4.8	4	28.6	1	14.3
	Below higher education	426	56.1	4	28.6	3	42.9
	Higher education	298	39.2	6	42.9	3	42.9
Family history of eye diseases	Neither	560	73.7	4	28.6	1	14.3
	Father	138	18.2	3	21.4	3	42.9
	Mother	43	5.7	6	42.9	2	28.6
	Both	30	4.0	2	14.3	0	0.0
Frequent eye inflammation	Yes	223	29.4	7	50.0	4	57.1
	No	537	70.6	7	50.0	3	42.9
Socioeconomic status	Sufficient	753	99.0	13	92.9	7	100.
	Insufficient	7	1.0	1	7.1	0	0.0
Physical activity	<2 hours/day	601	79.1	7	50.0	5	71.4
	>2 hours/day	159	20.9	7	50.0	2	28.6
School type	Private	46	6.3	2	14.3	1	14.3
	Governmental	714	93.7	12	85.7	6	85.7
Smartphone use	<2 hours/day	38	5.1	3	21.4	1	14.3
	2–4 hours/day	345	45.4	4	28.6	1	14.3
	>4 hours/day	377	49.5	7	50.0	5	71.4

The VI was significantly related to the gender of participants, with higher percentages of VI in females (p = 0.001), and to excessive smartphone use (p = 0.007). The ages and the type of school the participants were attending (p = 0.568 and 0.124, respectively) had no significant impact on the status of VI among the studied participants. The effect of various parameters on the status of VI is presented in Table 3.

**Table 3:** Effect of various parameters on the status of VI. BL = blindness; VI = visual impairment; SVI = severe visual impairment

Total participants = 781		No SVI/BL		SVI and BL		
		Number	%	Number	%	P-value
		760	%	21	%	
Sex	Female	374	49.2	15	71.4	0.001
	Male	386	50.8	7	28.6	
Age (years)	6–9	247	32.5	8	38.1	0. 568
	10–12	520	67.5	13	61.9	
Father’s education	Below higher education	283	37.2	13	61.9	0.02
	Higher education	477	62.8	8	38.3	
Mother’s education	Illiterate	36	4.8	5	23.3	0.001
	Below higher education	426	56.1	7	28.6	
	Higher education	298	39.2	9	25.5	
Family history of eye diseases	Neither	560	73.7	5	23.3	0.001
	Father	138	18.2	6	28.6	
	Mother	43	5.7	8	38.1	
	Both	30	4.0	2	9.5	
Frequent eye inflammation	Yes	223	29.4	11	52.4	0.023
	No	537	70.6	10	47.6	
Socioeconomic status	Sufficient	753	99.0	20	98.0	0.089
	Insufficient	7	1.0	1	2.0	
Physical activity	<2 hours/day	601	79.1	12	78.5	0.016
	>2 hours/day	159	20.9	9	21.5	
School type	Private	46	6.3	3	15.0	0.124
	Governmental	714	93.7	18	85.0	
Smartphone use	<2 hours/day	38	5.1	4	26.5	0.007
	2–4 hours/day	345	45.4	5	24.5	
	>4 hours/day	377	49.5	12	49.0	

## Discussion

A total of 781 children (403 (51.6%) females and 378 (48.4%) males) attending various primary schools in Arar were enrolled in this study. The ages of the children ranged from six to 12 years. In accordance with the WHO classification, SVI was determined in 14 (1.8%) while blindness was seen in seven (0.9%) children.

In this study, the prevalence of SVI and blindness was 1.8% and 0.9%, respectively, which is slightly higher than the VI (1.4% low vision and 0.04% blindness) estimated in Arar previously [13]. This difference could be because all participants in this study were exclusively school-going children and exposed to more study load and phone use, especially in the post-COVID-19 time.

The prevalence of SVI/blindness in children was reported to be 1.4% in the Republic of Suriname [14], 1.7% in Kenya [15], 0.68% in Nepal [16], and 3.8% in India [17]. On the other hand, a much higher prevalence of VI in children of 30% in Egypt [18], 22% in Pakistan [19], and 7% in Saudi Arabia [20] has been reported. These variations may be justified as this study focused exclusively on SVI and blindness while the other studies may have included mild VI as well. Moreover, the nature of the sample and the classification criteria used in the study also played a significant role.

This study revealed that VI was more common in children having excessive smartphone exposure. This statement is supported by a study that indicates an association of more visual problems with more digital exposure [21] and by the observations made in two systemic review meta-analyses [22-23].

In the current study, VI was significantly related to the gender of the participants, with higher percentages of VI in females. This is in line with the results reported in a very recent study conducted among Chinese school-going children [24]. The role of parents in eye health-seeking behavior for their children can play an important role in reducing childhood VI. There are unsatisfactory levels of knowledge among parents in Saudi Arabia about pediatric eye diseases [25].

Study limitations

As this study was cross-sectional, a causal relationship between tested independent variables and the dependent variable could not be determined. Moreover, collected data may be subjected to recall bias.

## Conclusions

This study confirms a high prevalence of SVI and blindness in school-going children in Arar City. Strategies focused on the planning of effective early screening and treatment modalities of the causes leading to such VI can help in reducing the burden of childhood VI. The findings provide information that indicates that screening for vision-related problems should be a priority at every health evaluation of school children. We also suggest that school teachers can play an important role by identifying the children with any kind of visual difficulty and referring them well in time to ophthalmologists for further care.
